# Effects of replacing alfalfa hay with *Atriplex nummularia* on the growth performance, digestibility, and energy usage of six-month old camel calves

**DOI:** 10.1186/s12917-026-05943-5

**Published:** 2026-10-15

**Authors:** Ahmed R. Askar, Ahmed A. Aman, Gamal A. Baraghit, Osama Raef, Elham M. Ghoneim, Faysal Fayed, Eman Saddick, Samir S. Abou El Ezz, Usama A. Nayel, Hashem H. Abd El Rahman, Sobhy Sallam, Mohsen M. Shoukry, Hamedi M. Kandil, Alaa E. Rabee

**Affiliations:** 1 Animal and Poultry Nutrition Department, Desert Research Center, Cairo, Egypt; 2 Animal Production Department, Faculty of Agriculture, Menoufia University, Menoufia, Egypt; 3 Animal and Poultry Physiology Department, Desert Research Center, Cairo, Egypt; 4 Animal Production Department, National Research Centre, Giza, Egypt; 5 Animal and Fish Production Department, Faculty of Agriculture, Alexandria University, Alexandria, Egypt

**Keywords:** Camels, *Atriplex*, Growth performance, Digestibility, Energy utilization

## Abstract

**Background:**

The use of halophytes as fodder, such as *Atriplex*, is an appropriate strategy for sustainable agriculture and a promising way to fill the gap in the camel feeding in arid and semi-arid areas. This experiment investigated the effects of feeding *Atriplex nummularia* instead of alfalfa hay on the growth performance, digestibility, and energy utilization in growing camel calves.

**Methods:**

Eighteen six-month-old male camel calves were employed and divided into three groups (*n* = 6). Animals received a concentrate feed mixture at 0.9% dry matter (DM) of their live body weight (BW) with different roughage sources at 1.1% DM of their BW. The roughages fed consisted of: alfalfa hay alone (T1), a 50:50 mixture of alfalfa hay and *Atriplex* (T2), or *Atriplex* alone (T3). During the growing study, animals were weighed once a week, and the proper concentrates and roughages were computed and applied immediately. At the end of the experiment, the digestibility trial, energy measurements, and rumen and blood metabolite analyses were conducted.

**Results:**

Replacing alfalfa hay with *Atriplex* significantly reduced total organic matter intake and digestibility (*P* < 0.01), resulting in lower BW changes and average daily gain with a poorer feed conversion ratio. Energy expenditure significantly differed among feeding regimens, with a greater energy balance for those fed alfalfa hay vs. *Atriplex*. A greater concentration of total protein, globulin, and urea was obtained in camels fed *Atriplex* (*P* < 0.05). The inclusion of the *Atriplex* in the diet decreased serum creatinine, cholesterol, and aspartate aminotransferase (*P* < 0.05). Replacing alfalfa hay with *Atriplex* resulted in lower rumen pH (*P* < 0.01) and higher total volatile fatty acids and ammonia levels (*P* < 0.05).

**Conclusions:**

In arid environments, *Atriplex nummularia* can partially or entirely replace alfalfa hay for growing camel calves; however, complete substitution resulted in significant reductions in average daily growth, nutrient digestibility, and feed efficiency. It proposed that *Atriplex* could be an effective camel fodder for enhancing drought resilience in desert environments.

## Introduction

The scarcity of animal feeds is encouraging the use of unconventional feed resources such as halophyte plants in arid and semi-arid regions [1]. These plants are an emerging solution to utilize marginal resources, such as saline soils and water, for providing animal feed [2]. Halophyte crops produce more biomass than traditional forages under saline cultivation conditions [2]. *Atriplex* has high salt and crude protein content as well as balanced amino acids and moderate crude fiber in comparison to high-quality forages [1, 3]. Halophytes also contain a high concentration of secondary metabolites such as phenols, tannins, flavonoids, and oxalate [4]. The moderate concentration of these metabolites confers beneficial effects as antipathogens, antioxidants, immunological stimulators, and antimicrobials [5]. Furthermore, these compounds protect the protein from rumen degradation while reducing rumen methanogens and methane production [5, 6]. However, excessive concentration of these metabolites reduces the palatability and rumen digestibility [4]. Therefore, raising well-adapted livestock, such as the dromedary camel, to use these halophytes is a viable strategy for sustainable agriculture [7].

Dromedary camels have unique physiological characteristics that enable them to withstand arid climates [8, 9] with high temperatures, poor vegetation, limited feed and water, and secondary metabolites in halophytes [8, 10]. Camels prefer to browse halophytes due to their high moisture and salt content, as they require more salt than other livestock species [11]. Furthermore, camel saliva contains mucin glycoproteins that protect the mucosa of the digestive tract from mechanical damage and prevent the negative effect of plant tannins on protein metabolism in the rumen [7, 11]. These facts explain camels’ high intake of *Atriplex* [3, 10]. Seasonal feed shortage is the main constraint in camel production, particularly during drought seasons. Therefore, unconventional sources of roughage, such as *Atriplex*, are being sought to address this issue [3].

The forage quality is one of the main constraints in animal nutrition; therefore, knowledge about forage quality and its interaction with animals’ performance is required to optimize its integration into animal feeding plans [4]. Rabee et al. [7] explained that anti-nutritional factors in saltbushes adversely impact the rumen microbiota and fermentation, which impacts the digestibility and performance. In the same line, Abdel-Wahed [3] reported a drop in the digestibility except for the crude protein in the growing camel calves fed *Atriplex* compared to clover hay and rice straw. Shawket et al. [11] found that including the *Atriplex* in camel diets had no effect on the digestibility of dry and organic matter but decreased the crude protein and crude fiber digestibility. Furthermore, including the *Atriplex* in the diet reduced cellulolytic enzymes, rumen ammonia, and total volatile fatty acids [4, 7]. Growing camels given *Atriplex* had comparable feed intake, digestibility, and growth performance to those clover hay-fed camels and superior to straw-fed camels [3, 10].

There is less information on incorporating saltbushes into intensive camel farming with its abilities to survive extreme weather conditions at a low feeding cost [12]. Furthermore, this study aimed to investigate the effect of replacing alfalfa hay with *Atriplex* on feed intake, growth performance, and digestibility in growing camel calves.

## Materials and methods

The experiment was carried out in the “National Campaign for the Promotion of Camel Productivity” farm, El-Shalateen Research Station, which belongs to Desert Research Center, Wadi-Hodien, El-Shalatein, Red Sea Governorate, about 1200 km from Cairo, Egypt at 22°00′ 7.20″ N and longitude 36°48′ 9.55″ E. It is an arid region with a desert environment that has almost no precipitation all year long.

### Animals and treatments

The current experiment utilized eighteen six-month-old male camel calves, weighing an average of 156 ± 7.81 kg. They were kept in half-shaded, sandy-floor pens with free access to drinking water in half-plastic barrels. The animals were sourced from the “National Campaign for the Promotion of Camel Productivity” farm, which belongs to the Desert Research Center, Egypt. The animals received a concentrate feed mixture (CFM) at 0.9% of their live body weight (BW), with roughage at 1.1% of their BW to cover the nutrient requirements for maintenance and growth [13].

Animals were divided into three groups (*n* = 6) to be fed one of three roughages: alfalfa hay alone (T1), a 50:50 mixture of alfalfa hay and *Atriplex* (T2), or *Atriplex* alone (T3), in which the tender part of the *Atriplex nummularia* was harvested, sun-dried for 10 days. The CFM and roughage were individually offered twice a day, at 08:00 and 14:00 h. The proximate analysis of CFM, alfalfa hay, and *Atriplex nummularia* is shown in Table 1. The study lasted for 145 days, including a 45-day adaptation period for feeding *Atriplex* to growing camels. The growing experiment began immediately after adaptation and lasted for 100 days, in which animals were weighed once a week, and the proper concentrates and roughages were computed based on their new weight and applied immediately. However, the digestibility trial, energy measurements, and rumen and blood metabolite analysis were conducted at the end of the growing experiment.

**Table 1:** Chemical composition of concentrate supplement, alfalfa and *Atriplex* based on a dry matter (DM) basis

Ingredients	*Concentrate feed	Alfalfa	Atriplex
Dry matter, g/ kg fresh matter	943	935	861
Gross energy, MJ/ kg dry matter	14.2	15.9	12.6
Chemical composition, g/ kg DM:			
Organic matter	887	881	748
Crude protein	141	127	118
Neutral detergent fiber	387	510	510
Acid detergent fiber	198	398	367

*The concentrate consisted of 55% corn, 15% soybean meal, 10% cottonseed meal, 15% wheat bran, 2.5% limestone, 1.5% salt, 0.5% sodium bicarbonate, 0.1% yeast, 0.1% antitoxins, and 0.3% premix

### Digestibility trial

Animals were fitted with fecal bags and given three days to adjust to their new surroundings before collecting feces for the next seven days. They were weighed at the beginning and end of the trial, and the amount of feed intake was recorded daily. Fecal bags were emptied twice a day, and total fecal output was then measured. A 10% subsample was taken and pooled in individual composite samples through the collection period. The samples were dried at 65 °C for 48 h and preserved with feed samples for future analysis. The digestibility was estimated using the acid-insoluble ash corrected for its fecal recovery [14] based on the following equations: (1) The amount of internal marker in feces = The amount of internal marker in intake. (2) Fecal output * %internal marker in feces = Total intake * %internal marker in intake. (3) Fecal output = (Total intake * %internal marker in intake) / %internal marker in feces. (4) Digestibility (%) = (Total intake – Fecal output) * 100/ Total intake. Please note that the total intake equals the sum of the known concentrate and forage intakes.

The rumen fluid and blood samples were collected 2–3 h after the morning feeding. Blood samples were obtained via jugular vein puncture using heparin as the anticoagulant and centrifuged at 10,000×g for 5 min and then stored at − 20 °C for the measurements of the total protein, albumin, urea, creatinine, cholesterol, triglyceride, aspartate aminotransferase (AST), and alanine aminotransferase (ALT) using the laboratory assay kits (Biodiagnostic, Giza, Egypt). Moreover, the rumen liquor samples were withdrawn via a stomach tube [13], and immediately a pH was recorded using a digital pH meter (WPA CD70, ADWA, Szeged, Hungary). Two samples were taken out and then frozen for later determining ammonia [15] and volatile fatty acid (VFA [16]), analysis.

### Energy expenditure

Askar [17] and Askar et al. [18] earlier described the calorimetry system and its use in small ruminants and camels, respectively. Experimental animals were outfitted with a face mask that allowed open-circuit respiration for monitoring O₂ consumption. The individual energy expenditure (EE)/heart rate (HR) ratio was determined by taking simultaneous readings from the RCX3 HR (Polar Electro Oy, Kempele, Finland). The concentration of O2 was determined with a fuel cell FC-1B O2 analyzer (Sable Systems, Las Vegas, NV), while EE was estimated by assuming a constant thermal equivalent of 20.47 kJ per liter O2 [19]. Moreover, the HR was thought to be individually collected at 1-minute intervals for at least 24 h, with the daily EE being calculated from the EE: HR ratio for each animal. The Polar software was employed to analyze the collected data.

### Weather data

The ambient temperature (T °C) and relative humidity (RH) were measured daily using RC-4HA Temperature and Humidity Data Logger and used to compute the temperature–humidity index (THI) as follows: (0.8 x T) + [(RH/100) x (T − 14.4)] + 46.4 [20].

### Chemical analyses

The AIA was used as an internal marker and analyzed according to the method of Van Keulen and Young [21]. Dried samples of feeds and feces were ground and analyzed according to the method of AOAC [15] to measure dry matter (DM), organic matter (OM), and crude protein (CP). Neutral detergent fiber (NDF) and acid detergent fiber (ADF) were measured using ANKOM Technology (ANKOM Technology, New York, United States [22]), . The gross energy (GE) was measured by bomb calorimeter (IKA, model C 200, Staufen, Germany). Metabolizable energy (ME) was considered as 82% of digestible energy (DE) intake.

### Statistical analysis

The data was statistically analyzed using the GLM procedure of the SAS statistical package [23]. One-way analyses of variance were employed with feeding treatment as a fixed effect and animal as the random effect. The model was Yij = µ + Ti + eij, where Yij is the dependent variable, µ the overall mean, Ti the fixed treatment effect, and eij is the residual. Differences among means were determined by least significant difference with a protected F-test, and differences with *P* < 0.05 were accepted as statistically significant.

## Results

**Fig. 1:**
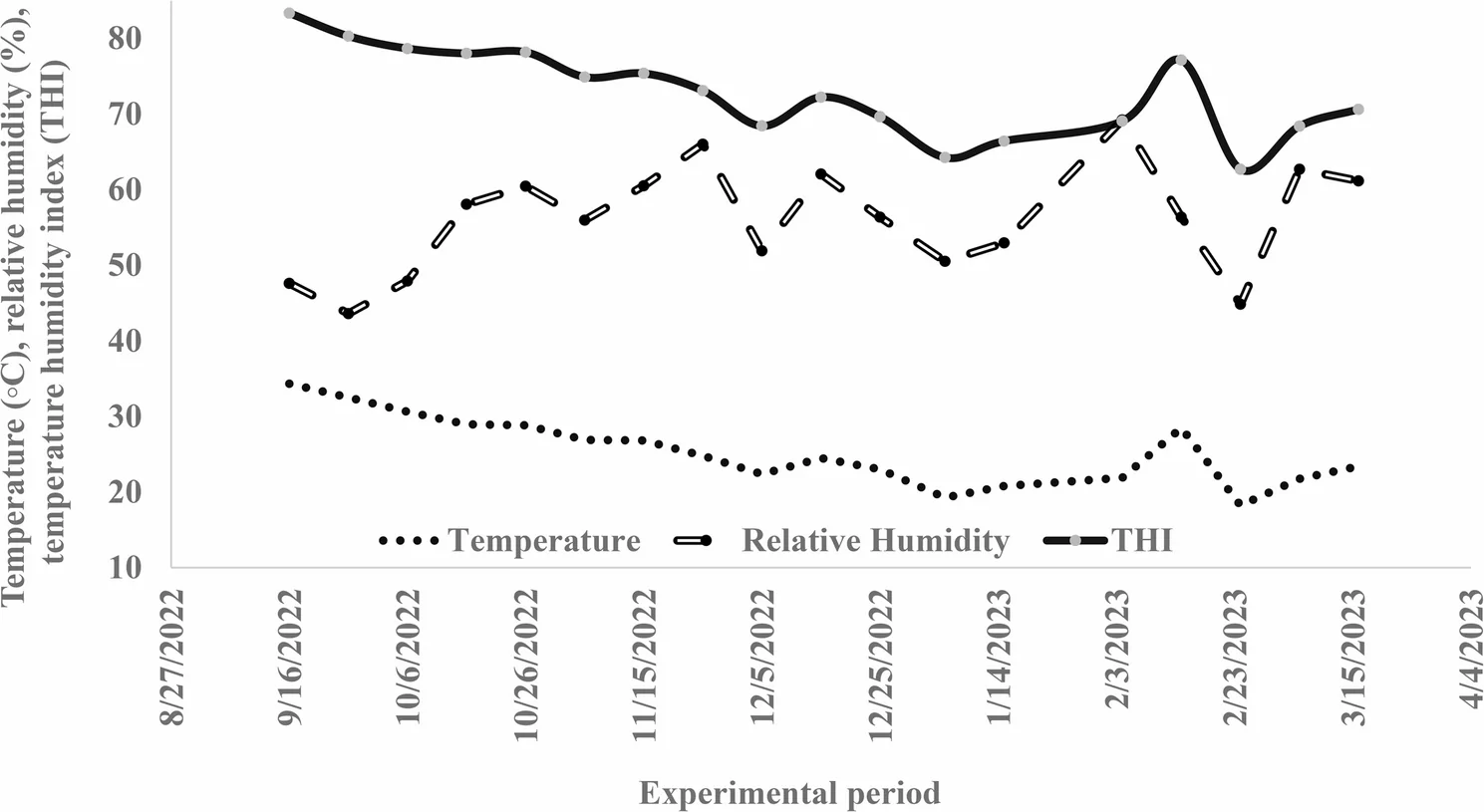
Mean temperature, relative humidity, and temperature–humidity index (THI) throughout the experimental period that growing camel calves were exposed to September–March

The mean ambient temperature was 25.4 °C, while those of RH and THI were 56.0 and 72.8%, respectively (Fig. 1).

### Feed intake and growth performance

The average initial BW was similar (*P* = 0.997) between camel groups (Table 2). Final BW in camel groups fed *Atriplex* (213 kg, T2 and 205 kg, T3) was numerically lower (*P* = 0.572) than that of those fed alfalfa hay (229 kg, T1) without a significant difference (Table 2). The average daily gain (ADG) was significantly (*P* < 0.01) declined by 18 and 29% in calves fed a mix of alfalfa and *Atriplex* (T2) and those fed *Atriplex* alone (T3), respectively, compared to animals fed alfalfa hay (T1) (Table 2). Total DM intake was similar among experimental groups. Furthermore, a poorer feed conversion ratio (FCR) was observed with camels fed *Atriplex* (7.46, T3) compared to those fed alfalfa hay (4.82, T1), while those fed a mix of alfalfa hay and *Atriplex* (6.34, T2) had an intermediate value (Table 2).

**Table 2:** Growth performance of growing camel calves fed *Atriplex* instead of alfalfa at different levels in the diet

Items	Concentrate feed at 0.9% of body weight				
	Alfalfa	Alfalfa/ Atriplex	Atriplex	SEM	*P*-value
Fattening period, days	100	100	100		
Initial body weight, kg	155	155	156	19.6	0.997
Final body weight, kg	229	213	205	20.6	0.572
Body weight changes, kg	74.0^a^	57.8^b^	48.4^b^	3.10	0.000
Average daily gain, g/ day	793^a^	572^b^	479^c^	32.5	0.000
Dry matter intake, g/ day:					
Concentrate	1694	1614	1589	172.7	0.847
Hay	2116^a^	1008^b^	0.00^c^	137.3	0.000
* Atriplex*	0.00^c^	1008^b^	1986^a^	132.2	0.000
Total roughage	2116	2017	1986	215.8	0.847
Total	3810	3631	3575	388.6	0.847
Dry matter intake, g/ kg BW^0.75^:					
Concentrate	32.8	32.2	32.2	0.80	0.713
Hay	41.0^a^	20.1^b^	0.00^c^	0.60	0.000
* Atriplex*	0.00^c^	20.1^b^	40.3^a^	0.64	0.000
Total roughage	41.0	40.2	40.3	0.99	0.694
Total	73.9	72.4	72.5	1.80	0.698
Feed conversion ratio	4.82^b^	6.34^a^	7.46^a^	0.592	0.005

^a, b,c^ Means having different superscripts within the same row differed significantly (*P* < 0.05). *SEM*  Standard error of means

### Digestibility of nutrients

Although total DM intake was similar among camel groups, total OM intake was adversely impacted (*P* < 0.01) when alfalfa hay was replaced with *Atriplex*, reflecting a significant decrease in the intake of CP and ADF (*P* < 0.05) (Table 3). Furthermore, camels fed alfalfa hay had higher (*P* < 0.01) digestibility, whereas those fed *Atriplex*, either alone (T3) or combined with alfalfa hay (T2), had significantly lower digestibility (Table 3).

**Table 3:** Feed intake and digestibility of growing camel calves fed *Atriplex* instead of alfalfa at different levels in the diet

Items	Concentrate feed at 0.9% of body weight				
	Alfalfa	Alfalfa/ Atriplex	Atriplex	SEM	*P*-value
Body weight, kg	229	194	190	23.1	0.237
Total nutrients intake, g/ day:					
Dry matter	4297	3633	3570	431.7	0.237
Organic matter	3798^a^	3084^ab^	2894^b^	374.2	0.086
Crude protein	573	476	458	57.1	0.154
Neutral detergent fiber	1953	1651	1622	196.1	0.237
Acid detergent fiber	1322	1088	1038	131.0	0.124
Total nutrients intake, g/ kg BW^0.75^:					
Dry matter	72.7	69.8	69.8	1.85	0.240
Organic matter	64.3^a^	59.3^b^	56.5^b^	1.60	0.001
Crude protein	9.69^a^	9.15^b^	8.94^b^	0.245	0.033
Neutral detergent fiber	33.0	31.7	31.7	0.84	0.245
Acid detergent fiber	22.4^a^	20.9^b^	20.3^b^	0.56	0.009
Apparent nutrients digestibility:					
Dry matter	72.5^a^	69.4^b^	64.0^c^	0.60	0.000
Organic matter	77.8^a^	75.3^b^	69.9^c^	0.54	0.000
Crude protein	71.6^a^	67.0^b^	60.3^c^	1.20	0.000
Neutral detergent fiber	61.0^a^	57.3^b^	49.0^c^	1.37	0.000
Acid detergent fiber	52.8^a^	47.4^b^	37.9^c^	1.54	0.000

^a, b, c^ Means without a common superscript letter in the row are differed (*P* < 0.05) between treatments. *SEM*  Standard error of means

### Energy utilization

The effects of replacing alfalfa hay with *Atriplex* on energy utilization are shown in Table 4. In line with OM intake and digestibility, increasing *Atriplex* in the diet reduced GE and DE (kJ/kg BW^0.75^) intakes. A higher DE (*P* < 0.01, %) was observed for those fed alfalfa hay vs. *Atriplex*, while those fed mixed roughage had an intermediate value. The ME followed the same pattern as the DE. However, HR (beats/min) significantly differed among treatments. Replacing alfalfa hay with *Atriplex* had a significant negative effect (*P* < 0.01) on EE, which was greatest for camel calves fed alfalfa and lowest for those fed *Atriplex*. This, together with higher ME intake, resulted in a greater energy balance for calves fed alfalfa hay vs. *Atriplex* as the sole forage.

**Table 4:** Energy utilization of growing camel calves fed *Atriplex* instead of alfalfa at different levels in the diet

Items	Concentrate feed at 0.9% of body weight				
	Alfalfa	Alfalfa/ Atriplex	Atriplex	SEM	*P*-value
Energy utilization:					
Gross energy, kJ/ kg BW^0.75^	1100.5^a^	996.9^b^	929.8^c^	27.17	0.0001
Digestible energy, kJ/ kg BW^0.75^	813.5^a^	685.5^b^	577.4^c^	21.02	0.0000
Digestible energy, %	73.9^a^	68.9^b^	62.2^c^	0.86	0.0000
Metabolizable energy, kJ/ kg BW^0.75^	667.1^a^	562.1^b^	473.4^c^	17.24	0.0000
Heart rate, beat/ min	65.7^a^	56.2^b^	52.1^c^	1.06	0.0000
Energy expenditure, kJ/ kg BW^0.75^	433.5^a^	371.1^b^	344.1^c^	6.98	0.0000
Energy balance, kJ/ kg BW^0.75^	233.6^a^	191.0^b^	129.4^c^	17.46	0.0003

^a, b, c^ Means without a common superscript letter in the row are differed (*P* < 0.05). *SEM*  Standard error of means

### Blood and rumen metabolites

The effects of replacing alfalfa hay with *Atriplex* on some blood and rumen metabolites are shown (Table 5). A greater concentration of total protein, globulin, and urea was obtained in camels fed *Atriplex* (*P* < 0.05). *Atriplex* feeding decreased serum creatinine, cholesterol, and AST (*P* < 0.05). However, replacing alfalfa hay with *Atriplex* decreased rumen pH (*P* < 0.01) and increased total VFA and ammonia (*P* < 0.05) (Table 5).

**Table 5:** Blood and rumen metabolites of growing camel calves fed *Atriplex* instead of alfalfa at different levels in the diet

Items	Concentrate feed at 0.9% of body weight				
	Alfalfa	Alfalfa/ Atriplex	Atriplex	SEM	*P*-value
Blood parameters:					
Total protein, g/ dl	5.46^b^	5.20^b^	5.91^a^	0.193	0.009
Albumin, g/ dl	3.66	3.40	3.39	0.183	0.337
Globulin, g/ dl	1.81^b^	1.82^b^	2.51^a^	0.155	0.001
Cholesterol, mg/ dl	125	114	110	10.0	0.373
Triglyceride, mg/ dl	83.2^b^	85.9^ab^	89.2^a^	2.59	0.148
Urea, mg/ dl	4.03^b^	4.67^a^	4.51^a^	0.202	0.028
Creatinine, mg/ dl	1.84^a^	1.67^b^	1.39^c^	0.046	0.000
AST, IU/ dl	103^ab^	108^a^	96^b^	4.2	0.051
ALT, IU / dl	42.2	42.4	42.7	0.29	0.322
Rumen parameters:					
pH	6.77^a^	6.29^b^	5.83^c^	0.077	0.000
Ammonia, mg/dl	9.8^b^	14.7^a^	15.8^a^	1.61	0.032
Volatile fatty acids, m.eq/ dl	6.48^b^	9.93^a^	12.6^a^	1.29	0.007

^a, b, c^ Means without a common superscript letter in the row are differed (*P* < 0.05). *SEM*  Standard error of means

## Discussion

The limited availability of natural resources highlights the camel as an integral animal in sustainable agriculture because of its ability to convert halophytes, which other domestic animals do not prefer, into animal products [2, 7]. Halophytes chemical composition limits their palatability and ingestion [24]. The results obtained for the chemical composition indicated that *Atriplex* has lower OM and CP and similar fiber fraction contents compared to alfalfa, which is similar to that reported by Farid et al. [10], while that of alfalfa hay is within the range indicated by Askar et al. [18]. However, *Atriplex* contains several secondary metabolites, such as phenols, tannins, flavonoids, saponins, oxalates, alkaloids, and resins [7].

### Digestibility and performance

The secondary metabolites in halophytes affect rumen microbial population, digestibility, and performance [7, 12], which might explain the lower nutrients digestibility and lower ADG in calves fed *Atriplex* (T2 and T3) compared to those fed alfalfa hay (T1). Abdel-Wahed [3] reached a similar conclusion, reporting declines in nutrient digestibility with the exception of CP digestibility, which improved when alfalfa was replaced by *Atriplex*. Shawket et al. [11] found decreased digestibility of CP, CF, and fat in camels fed *Atriplex*. Askar et al. [25] reported a drop in digestibility when sheep and goats were fed *Atriplex* instead of alfalfa hay. Dadvar et al. [4] concluded that antinutritional factors in *Atriplex* negatively affected the rumen microorganisms. Rabee et al. [7] reached similar findings and reported that tannins inhibited camel rumen cellulolytic bacteria such as *Fibrobacteres*, which negatively impacted DM and NDF digestibility in the rumen. These findings are supported by a reduction in cellulolytic enzymes in camels fed *Atriplex* [4].

The reduction in digestibility caused by replacing alfalfa hay with *Atriplex* was associated with a decrease in ADG (T2 and T3). This decline in growth rate as a result of feeding *Atriplex* was also reported in lambs when wheat straw was completely replaced by *Atriplex* [26]. In contrast, Farid et al. [10] and Abdel-Wahed [3] found that substituting *Atriplex* for clover hay and rice straw improved the ADG of camel calves, which could be attributed to the *ad libitum* feeding of fresh chopped *Atriplex* compared to restricted dried crushed *Atriplex* (1.1% of BW, Tables 2 and 3). Shawket et al. [11] indicated that digestibility and growth rate were improved in camels fed *Atriplex* when the feeding period was prolonged from 180 to 360 days, which might be attributed to the adaptation of rumen microorganisms to the antinutritional factors [10, 11], highlighting the camel as a well-adapted animal to feed on halophytes. However, the growth rate in the current study (Table 2) was greater than that of camel calves fed alfalfa hay or *Atriplex* [3, 10, 11], most likely due to the sex, age, or initial BW of the camel calves [13]. Also, the breed may be involved [27, 28], as this growth rate was greater than that of camel calves fed alfalfa or *Alhagi maurorum* [12], with higher DM intake than in the current study. However, the inclusion of the *Atriplex* did not impact the feed intake (g/kg BW^0.75^, Tables 2 and 3), which was within the normal range of camel calves fed different forages [3, 10, 11, 13, 18]. Previous studies on camel calves [3, 10] reported that including the *Atriplex* in camel diets increased the feed intake compared to camels fed alfalfa hay.

### Rumen fermentation and blood metabolites

The replacement of alfalfa hay with *Atriplex* increased rumen ammonia, resulting in greater total protein and urea levels in blood. In contrast, Abdullah et al. [26] observed a decrease in rumen ammonia in lambs fed *Atriplex*, which was similar with the findings of Dadvar et al. [4] in camels. They attributed the decreased ammonia to the lower nitrogen degradation in the rumen due to the presence of tannins in the *Atriplex* [3, 4]. However, camel calves, in the current study, were fed concentrate first, followed by *Atriplex*, which was fed at a slower rate than alfalfa hay. This concentrate feed consisted of 14.1% CP, including different sources of protein with a considerable amount of degradable nitrogen, which probably contributed to the elevated ammonia levels in the rumen. Rabee et al. [29] recently reported a significant decline in rumen ammonia in camel calves fed *Atriplex* rather than alfalfa hay for 6 months longer than those in the current experiment, highlighting the camel’s adaptation to feeding on halophytes with a similar rate to alfalfa hay. Shawket et al. [11] found that feeding *Atriplex* for 12 months rather than 6 months enhanced camel calves’ digestibility and growth rate, which was attributed to the rumen microorganisms adapting to *Atriplex*’s antinutritional factors. On the other hand, a higher VFA when alfalfa hay was replaced with *Atriplex* coincided with a lower rumen pH. A considerable amount of non-structural carbohydrates in the concentrate feed with 55% corn was probably the main reason for the lower pH and higher VFA when *Atriplex* was fed instead of alfalfa (T2 and T3). Abdullah et al. [26] noticed an increase in rumen VFA in lambs fed *Atriplex*. However, higher rumen concentrations would certainly meet the requirements for microbial synthesis [30], implying higher nitrogen degradation in conditions of high fermentable energy availability (higher VFA), which would enhance microbial protein synthesis, nutrient utilization (Table 3), and, as a result, camels’ performance (Table 2). In contrast, total VFA numerically decreased in camels fed *Atriplex*, as previously noted in growing camels fed *Atriplex* or camelthorn [4, 12]. Similarly, Ahmed et al. [6] found a decline in rumen VFA in lambs fed *Atriplex*, which could be attributable to diluted ruminal content due to increased water consumption when animals were fed saltbushes, but this was not relevant to the current study with camel calves. Rabee et al. [29] recently reported that feeding *Atriplex* to camel calves for an extended period of time resulted in a similar total VFA.

The blood metabolites were within the normal range for growing camels [12, 13, 31], but differed from those reported by Faraz [32] and Faraz et al. [33], implying that breed or age may be a factor. The replacement of alfalfa with *Atriplex* increased blood total protein, which is consistent with the findings in lambs fed *Atriplex* [6]. Furthermore, a higher blood urea was observed when *Atriplex* rather than alfalfa hay was fed. The findings were in line with Dadvar et al. [4] and could be attributable to increased ammonia and its absorption through the rumen wall [4, 6]. However, the decline in blood cholesterol and liver enzymes (AST) was linked to a lower rumen pH, and also reported in camel calves after replacing alfalfa with *Alhagi maurorum* [12]. Ahmed et al. [6] reported a decline in blood cholesterol and an increase in blood urea in lambs fed *Atriplex*. Ghavipanje et al. [12] reported that active compounds in the halophytes, such as flavonoids and phenols, decrease blood cholesterol, liver enzymes, triglycerides, and oxidative stress. Ahmed et al. [6] explained that tannins in the *Atriplex* form a complex with fatty acids, which decreases the absorption of cholesterol and increases the fat excretion. The decline in the liver enzymes indicated normal liver function in camels fed *Atriplex* [6, 12].

### Energy utilization

The relationship between EE and ME intake revealed that *Atriplex* had significantly higher EE than alfalfa hay (EE = 73 vs. 65% of ME intake, respectively), which could be explained in part by the effects of the forage quality on gut mass and energy use [25]. It is expected that *Atriplex*, as a low-quality forage, requires more energy for digestion than alfalfa hay, which is a high-quality forage; this difference is likely to contribute to the variation in EE between the two forage types. This, together with the lower ME intake for *Atriplex* vs. alfalfa hay, was reflected in the energy balance, which was greater for those fed alfalfa hay vs. *Atriplex*, while the mixed forage treatment had an intermediate value (Table 4), consistent with the lower ADG for *Atriplex* vs. alfalfa hay (Table 2). Similar findings were observed with sheep and goats when they were fed *Atriplex nummularia* compared to alfalfa hay [17]. Forage quality has been reported to affect EE and requirements in livestock species [34, 35]. Although *Atriplex* contains various secondary metabolites that reduce its quality [7] and affect its EE, it has a positive energy balance with a moderate digestible energy (62.2%, Table 4) and moderate ADG (480 g/day, Table 2). This is considered a positive result with feeding *Atriplex*, although camel calves fed alfalfa hay performed better (Table 2).

Halophytic plants were reported to have varied degrees of digestion and use among animal species. Small ruminants fed on *Atriplex* cannot sustain their live BW [36–38]. Sheep and goats fed *Atriplex nummularia* [39] or *Acacia saligna* [40] had a negative impact on feed intake with a negative energy balance, but those fed alfalfa hay had a positive balance. However, the energy supplement (concentrate feed, Table 1) might have great importance in improving performance when *Atriplex* was fed. *Atriplex* could prove to be a good and cheap source of nitrogen [17, 41, 42] in the presence of energy sources. Ben Salem et al. [38] reported that *Atriplex* is deficient in energy and has approximately 65% of nitrogen as non-protein nitrogen. In the current study, replacing *Atriplex* with alfalfa did not affect the forage or the total intake (Tables 2 and 3), with a positive energy balance (Table 4) and moderate or good performance (Table 2). Camels can handle high salt levels and adapt to water scarcity and oxalate because of efficient kidney and rumen adaptations, while small ruminants are more sensitive to high ash/salt loads [25, 40, 43], and their intake is limited by high salt and secondary compounds [25, 44]. Excessive intake of *Atriplex* with high salt levels in small ruminants may reduce palatability and overall consumption, potentially leading to the risk of oxalate poisoning or digestive disturbance [41, 43]. Water requirements increase when feeding *Atriplex*, as animals need to excrete salts; however, camels can tolerate long intervals without water [13], while small ruminants require frequent access to water when fed *Atriplex* due to increased water demand [41].

Generally, feeding *Atriplex* to camels is safe, although excessive amounts may soften feces [44] as a result of consuming a large amount of water. *Atriplex* is more palatable and preferred by camels due to their high moisture and electrolyte content [10, 45, 46]. On the one hand, it may be related to their greater need for salt than other animal species, which is in higher proportion in the *Atriplex*. Camels require six to eight times as much salt as other animals [47, 48], with camels without regular access to salty feed requiring approximately 140 g of salt per day [49]. On the other hand, camel saliva contains varying amounts of high molecular weight mucin glycoprotein, which protects the mucosa of the digestive tract from mechanical injuries and fixes plant tannins, preventing their negative effects on protein metabolism in the rumen [50]. It is concluded that *Atriplex* is considered a strategic forage for enhancing drought resilience; it can be efficiently utilized by camels, supporting maintenance and moderate production, and serves as a valuable source of protein and mineral supplements [44].

## Conclusion

In arid regions where cattle cannot be raised, *Atriplex nummularia* can partially or entirely replace alfalfa hay for growing camel calves; however, complete substitution resulted in significant reductions in average daily growth, nutrient digestibility, and feed efficiency. It suggested that *Atriplex* could be an effective camel fodder for enhancing drought resilience in desert environments.

## Data Availability

All data and materials are owned by the authors and/or no permissions are required.

## Abbreviations

DM: Dry matter
BW: Body weight
CFM: Concentrate feed mixture
AST: Aspartate Aminotransferase
ALT: Alanine Aminotransferase
VFA: Volatile fatty acids
HR: Heart rate
EE: Energy expenditure
RH: Relative humidity
T: Ambient temperature
THI: Temperature–humidity index
OM: Organic matter
CP: Crude protein
NDF: Neutral detergent fiber
ADF: Acid detergent fiber
SEM: Standard error of means
ADG: Average daily gain
FCR: Feed conversion ratio
GE: Gross energy
DE: Digestible energy
ME: Metabolizable energy
